# Chromosome aberrations in pressure-induced triploid Atlantic salmon

**DOI:** 10.1186/s12863-020-00864-0

**Published:** 2020-06-06

**Authors:** K. A. Glover, A. C. Harvey, T. J. Hansen, P. G. Fjelldal, F. N. Besnier, J. B. Bos, F. Ayllon, J. B. Taggart, M. F. Solberg

**Affiliations:** 1 Institute of Marine Research, Bergen, Norway; 2 Department of Biological Sciences, University of Bergen, Bergen, Norway; 3 ZEBCARE, Nederweert, The Netherlands; 4 University of Stirling, Stirling, Scotland

**Keywords:** Triploid, Trisomy, Aneuploid, PUD, Mosaic, Aquaculture, Environmental impact, Welfare

## Abstract

**Background:**

Triploid organisms have three sets of chromosomes. In Atlantic salmon, hydrostatic pressure treatment of newly fertilized eggs has been extensively used to produce triploids which are functionally sterile due to their unpaired chromosomes. These fish often perform poorly on commercial farms, sometimes without explanation. Inheritance patterns in individuals subjected to pressure treatment have not been investigated in Atlantic salmon thus far. However, work on other species suggests that this treatment can result in aberrant inheritance. We therefore studied this in Atlantic salmon by genotyping 16 polymorphic microsatellites in eyed eggs and juveniles which had been subjected to pressure-induction of triploidy. Communally reared juveniles including fish subjected to pressure-induction of triploidy and their diploid siblings were included as a control.

**Results:**

No diploid offspring were detected in any of the eggs or juveniles which were subjected to hydrostatic pressure; therefore, the induction of triploidy was highly successful. Aberrant inheritance was nevertheless observed in 0.9% of the eggs and 0.9% of the juveniles that had been subjected to pressure treatment. In the communally reared fish, 0.3% of the fish subjected to pressure treatment displayed aberrant inheritance, while their diploid controls displayed 0% aberrant inheritance. Inheritance errors included two eyed eggs lacking maternal DNA across all microsatellites, and, examples in both eggs and juveniles of either the maternal or paternal allele lacking in one of the microsatellites. All individuals displaying chromosome aberrations were otherwise triploid.

**Conclusions:**

This is the first study to document aberrant inheritance in Atlantic salmon that have been subjected to pressure-induction of triploidy. Our experiments unequivocally demonstrate that even when induction of triploidy is highly successful, this treatment can cause chromosome aberrations in this species. Based upon our novel data, and earlier studies in other organisms, we hypothesize that in batches of Atlantic salmon where low to modest triploid induction rates have been reported, aberrant inheritance is likely to be higher than the rates observed here. Therefore, we tentatively suggest that this could contribute to the unexplained poor performance of triploid salmon that is occasionally reported in commercial aquaculture. These hypotheses require further investigation.

## Background

A triploid organism has three haploid chromosome sets. When induced in a species of vertebrate where this has not developed as an evolutionary strategy, it typically renders the individual sterile, with males producing aneuploid sperm and females displaying gonadal dysgenesis [1–3]. Within fish, spontaneous triploidy has been documented at low frequencies in cultured populations [4–6], and has even been very occasionally observed in otherwise diploid wild populations [7]. In some species of fish, for example in the crucian carp species complex (*Carassius auratus*), both diploid and triploid forms exist in the wild, whereby the triploid form reproduces by gynogenesis [8, 9]. Triploidy can also be deliberately induced in fish and shellfish using a variety of approaches [2, 10, 11], although for most species the current method of preference is a hydrostatic pressure treatment of eggs shortly after fertilization that leads to retention of the second polar body. The result is a triploid offspring with two sets of chromosomes from the dam and one from the sire.

Atlantic salmon (*Salmo salar*) is the economically most significant species of fish in global aquaculture. Within this industry, triploids have and continue to be extensively used [12, 13]. This is primarily to produce a sterile salmon to mitigate further genetic interactions between domesticated escapees and wild conspecifics, a situation that represents one of the greatest long-term challenges to an environmentally sustainable salmonid aquaculture industry [14]. However, despite substantial efforts to commercialize triploid Atlantic salmon aquaculture, most global Atlantic salmon production is still based on rearing diploid fish. Although there are many reasons for this, it is in part because triploid salmon have, at least occasionally, proved challenging to rear on a commercial scale [12, 13, 15]. An underlying factor is that the biology and physiology of triploid salmon is different to their diploid siblings [2, 16], however, in some circumstances the reasons underpinning poor performance, including high mortalities, are not always clear [13, 15].

The most commonly used method for detection of triploid fish is analysis of blood cells with flow cytometry [12]. Where it has been reported, the frequency of flow cytometry determined triploidy is typically high or very high in experimental batches. For example, based on an intensive investigation in coho salmon (*Oncorhynchus kisutch*), typical triploid frequencies were 97.0–99.8% [17]. In another study, examination of 10 offspring from each of 86 Atlantic salmon families gave 100% triploidy [18]. Other examples of high or very high frequencies of triploidy induction in experimental batches can also be seen in brook trout (*Salvelinus fontinalis*) [19], landlocked and anadromous Atlantic salmon and their hybrids [20] as well as interspecific hybrids between brook, brown (*Salmo trutta*) and rainbow trout (*Oncorhynchus mykiss*) [21]. However, under “sub-optimal” induction conditions, the frequency of triploid offspring drops, sometimes drastically [22, 23]. Furthermore, and rather significantly, hydrostatic-induction of triploid Atlantic salmon, as determined by flow cytometry, has been reported as low as 44% in batches of fish produced under commercial conditions [13].

Genotyping highly polymorphic microsatellites has also been used to determine ploidy in fish and insects [4, 5, 17, 24–27]. This approach has also been validated against flow cytometry in several species [5, 27, 28]. After pressure treatment, “failed-triploids” may first appear as normal diploids when investigated with flow cytometry [17]. However, pressure treatment is known to cause chromosome fragmentation and aneuploidy [29], and, as revealed in a study of coho salmon, at least some of the flow cytometry identified diploid individuals, i.e., the “failed-triploids”, were actually hyperdiploid aneuploids (i.e., between diploid and triploid) that lacked paternal DNA in some markers [17].

Despite the fact that triploid production has and continues to be extensively trialed in commercial Atlantic salmon production [12, 13, 15], and flow cytometry has demonstrated that occasional batches of fish reared on these farms have very low triploid induction rates [13], thus far, inheritance patterns in offspring that have been subjected to pressure treatment to induce triploidy have not been investigated in Atlantic salmon. Therefore, we conducted three interlinking experiments whereby microsatellite DNA profiling was used to investigate inheritance patterns in a pedigree material of pressure-induced triploid and diploid controls.

## Results

**Fig. 1:**
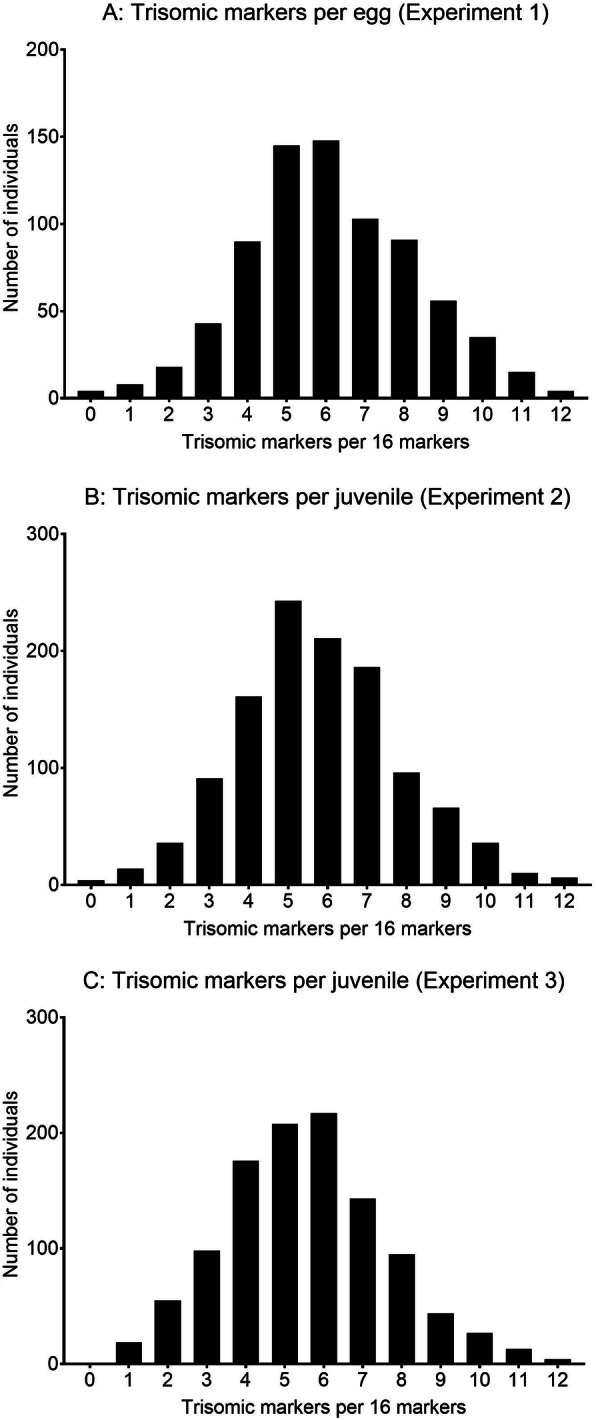
**a**/**b**/**c**. Numbers of microsatellites, out of the 16 polymorphic markers, displaying three alleles (trisomy) in fish treated with hydrostatic pressure to induce triploidy. Experiment 1; 760 eggs (**a**), experiment 2; 1161 juveniles (**b**) and experiment 3; 1099 juveniles (**c**)

Based upon the composite genotypes at 16 polymorphic microsatellite loci, no diploid offspring were observed among the 760 eggs nor 1161 juveniles that had been subjected to pressure-treatment in experiments 1 and 2 respectively (Fig. 1a/b, Supplementary File 1). In experiment 3, it was not possible to accurately evaluate the effectiveness of the pressure treatment. This is because the 1219 diploid controls were reared in the same replicate tanks as the 1099 pressure-induced triploids, and potential misidentifications as described in the methods, however infrequent, could theoretically occur. However, and importantly, as the triploids in experiment 3 arose from the exact same families and treatment-batches as those used in experiments 1 and 2, this was not expected to represent a challenge based on the results described above. Therefore, where it was possible to accurately report it in this study, pressure-induction of triploidy was highly effective (Supplementary File 1).

The median number of microsatellites displaying trisomy was 6, 5 and 6 for the eggs and juveniles in experiments 1, 2 and 3 respectively (Fig. 1a/b/c). The numbers of trisomic markers per individual were normally distributed, with no individuals displaying more than 12 trisomic markers, and very few displaying less than 2 trisomic markers. Eight of the offspring from experiments 1 and 2 had no trisomic markers which could indicate that these were diploid. However, the genotypes of these individuals were manually triple checked in Genemapper, and the allele amplification showed that these were triploid due to the greater amplification of the longer allele (thus suggesting 3 alleles) in some of the markers. This is however with the exception of two haploid eggs discussed below.

**Fig. 2:**
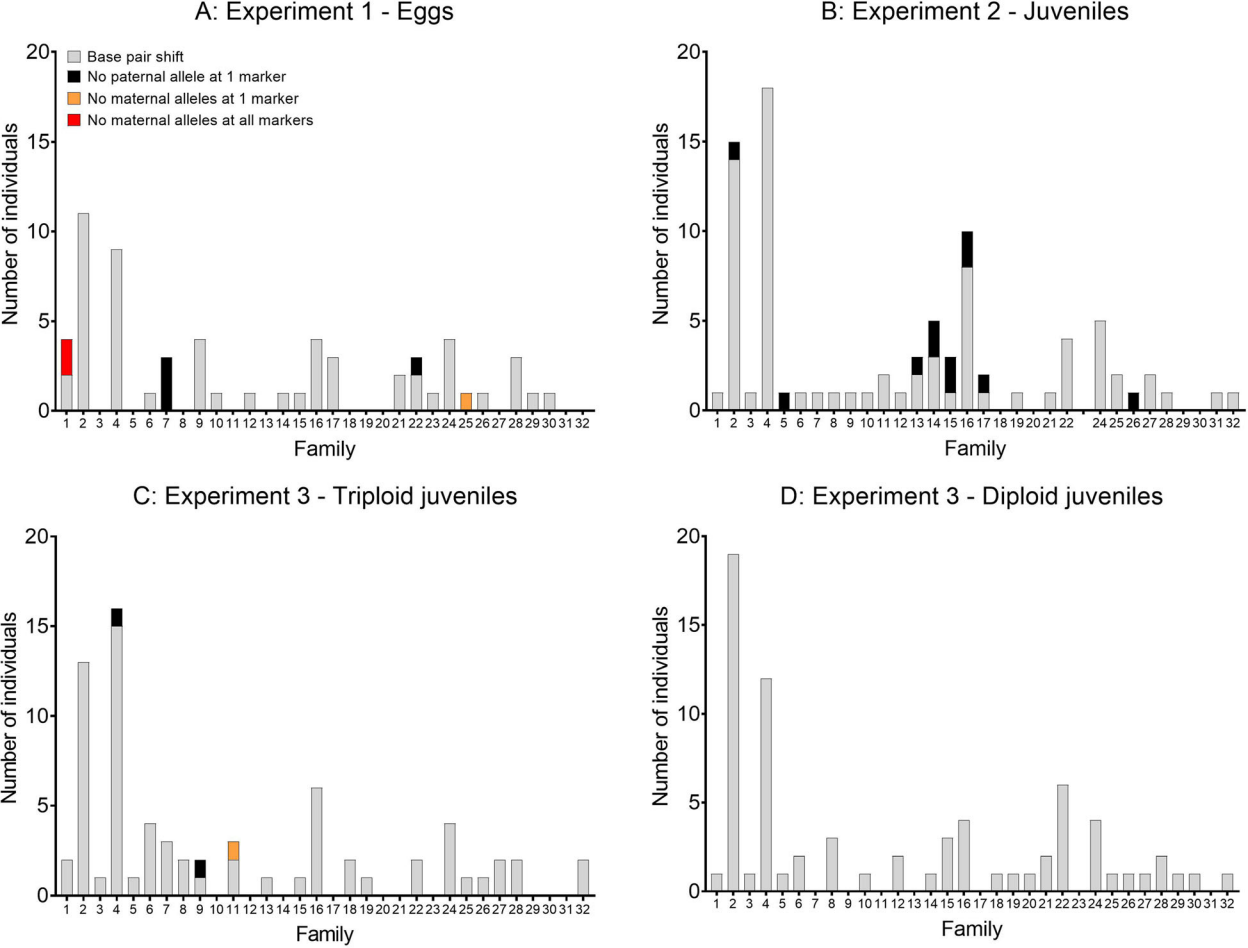
**a**/**b**/**c**/**d**. Numbers of inheritance errors in experiment 1; 760 eggs (A), experiment 2; 1161 juveniles (**b**), experiment 3; 1099 triploid juveniles (**c**) and 1219 diploid juveniles (**d**) displayed by type of error and by family

A total of 60 eggs (7.9%) in experiment 1, 86 juveniles (7.4%) in experiment 2, 73 of the diploid-control juveniles (6%) in experiment 3, and 72 pressure-treated juveniles (6.6%) in experiment 3 displayed inheritance abnormalities (Fig. 2a-d). In total, four different types of inheritance errors, that were non-randomly distributed among families, were reported. The great majority of these errors involved slippage of the microsatellite repeat motif. I.e., the offspring inherited an allele that had mutated from for example 200 bp to 204 bp or 196 bp in a microsatellite with a 4 bp motif. Mutations in the repeat motif are both highly common and previously very well documented in microsatellites [30–32], and not likely to be linked to the pressure-treatment itself. This is also supported by the very similar frequencies between the treatment and control groups as reported above. Therefore, these forms of inheritance errors were ignored further.

**Fig. 3:**
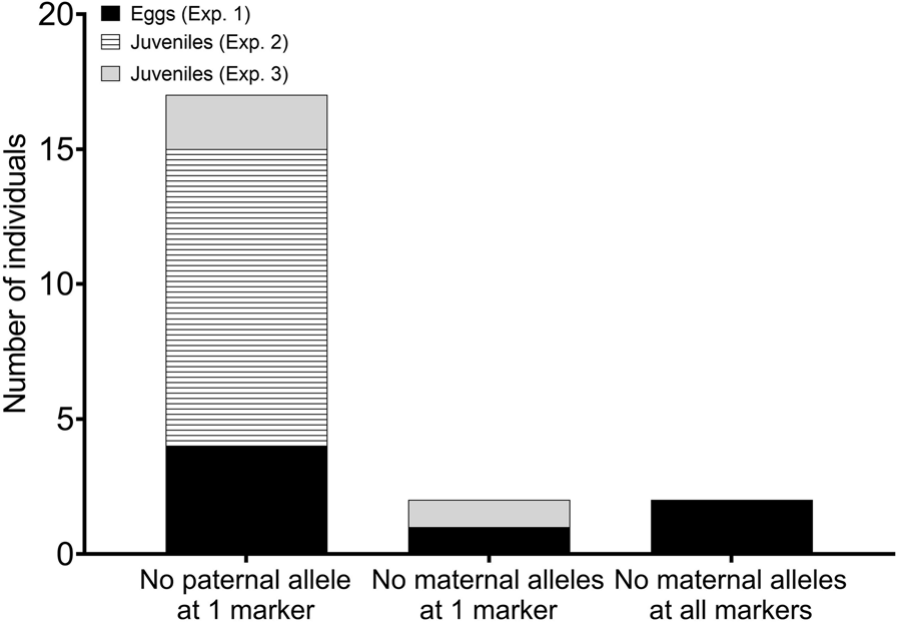
Total numbers (21) of individuals displaying inheritance errors observed in experiment 1 (760 eggs), experiment 2 (1161 juveniles) and experiment 3 (1099 juveniles) when excluding slippage in the microsatellite repeat

Excluding mutations in the repeat motif, inheritance errors were observed in a total of 21 individuals across all three experiments (Fig. 2a-c, Fig. 3). Significantly, these were only observed in the offspring that had been subjected to pressure-induction of triploidy. These inheritance errors were observed in the pressure-induced triploids in all three experiments but were non-randomly distributed among families (Fig. 2a-c). Of the three types of non-repeat motif slippage errors observed, all were observed in eggs, while two of them were observed in juveniles (Fig. 3, Supplementary File 1). Within both eggs and juveniles, two of the types of errors involved a single microsatellite where either the paternal allele or the maternal alleles were absent in the offspring’s genotype (Fig. 3, Supplementary File 1). Within eggs, another type of inheritance error was observed; namely, two eggs that completely lacked maternal alleles across all 16 microsatellites (Supplementary File 1). Both of these eggs originated from family 1.

Of the 5 eggs and 14 juveniles that lacked a maternal or paternal allele at one of the microsatellites, all but two individuals displayed three alleles at several or more microsatellites suggesting that they were almost certainly otherwise triploid. The two exceptions were re-checked for all markers and greater amplification of the longer allele was evident for several markers, strongly suggesting that these individuals were otherwise triploid or at least partially triploid.

## Discussion

To our knowledge, this is the first study to investigate microsatellite inheritance patterns in pressure-induced triploid Atlantic salmon. When applied correctly post-fertilisation, this treatment causes retention of the second polar body, thus producing an offspring with two sets of chromosomes from the dam, and one from the sire. Based upon this inheritance principle, and the fact that no normal diploid offspring were identified in the treatment groups where this could be precisely controlled for, the overall success of triploid induction in the present study was very high. Despite the success of treatment, we still detected multiple examples of three types of inheritance errors associated with the protocol, i.e., chromosome aberrations (excluding repeat motif slippage). These included two eyed eggs displaying only a single paternal allele across all markers, thus most likely lacking all maternal DNA, and a total of 19 cases in both eggs and juveniles that displayed either incomplete retention of paternal or maternal DNA at one of the microsatellites but were otherwise triploid. In contrast, no inheritance issues, excluding slippage of the microsatellite repeat motif, were reported in the diploid controls. Consequently, our data show for the first time in Atlantic salmon that triploid offspring may display diverse chromosome aberrations resulting from this treatment. Although our study has not demonstrated this, we tentatively hypothesize that low triploid induction, as reported occasionally in the aquaculture industry [13], may well lead to high frequencies of individuals displaying diverse chromosome aberrations. If this is the case, then this may contribute to the poor, and occasionally very poor performance of triploids in commercial aquaculture. This requires further examination.

### Inheritance errors and chromosome aberrations

It has been known for a long time that hydrostatic pressure treatment can lead to chromosome fragmentation and aneuploidy [29]. In the present study, 7.2% of the eggs and fish subjected to pressure-induction of triploidy, and 6% of the diploid controls, displayed inheritance errors. The great majority of these errors most likely reflected slippage of the repeat motif in the microsatellite. I.e., the maternal or paternal-derived allele was either one repeat motif longer or shorter in the offspring. These errors were almost certainly caused by slippage-mutations in the germ line of the parent and are therefore not linked with the pressure treatment itself nor failed triploid induction per se. This is supported in our results as the frequencies for this type of mutation were similar in the diploid controls and pressure-inducted triploid groups. Microsatellites display high mutation rates, and slippage of the repeat motif is highly common and very well described [30–32]. This specific observation in our data set is therefore not novel, not linked to the treatment, and therefore not considered further.

Two of the eggs, originating from a single family, displayed a striking lack of maternal DNA across all 16 informative markers and evidence of only one paternal allele (i.e., haploid or double haploid). No such observation was made in the juvenile fish, and we suspect these eggs would not have developed much beyond the sampling time point. It is not clear what underlying mechanism caused this extraordinary observation. However, a very similar phenomena, termed as (mosaic) paternal uniparental diploidy (PUD), has been reported for a low number of cases in humans [33–35]. In humans, this condition is fatal unless it is only observed in certain tissues and cells, i.e., as the mosaic form. Here, we isolated DNA from part or whole embryos and our results therefore indicate that our putative cases of PUD affected the entire organism which almost certainly would have resulted in fatality also. There are four known mechanisms driving mosaic PUD in humans, including meiotic and/or mitotic events [33, 34]. Which of these was responsible in our cases remains unresolved.

We detected a total of 19 eggs and juveniles that had been subjected to pressure treatment and were lacking a maternal or paternal allele at one of the markers. All appeared otherwise triploid, and thus would have been reported as normal triploids based on flow cytometry analysis. Incomplete paternal chromosome retention has previously been reported in coho salmon subjected to pressure treatment and initially identified as diploid by flow cytometry [17]. In all of their fish displaying complete or partial lack of paternal alleles, these authors observed that both maternal alleles were present in all cases. I.e., both polar bodies were retained. Thus, they suggested that in their observed cases, either the paternal chromosomes failed to transmit from the sperm during fertilization, or alternatively, paternal chromosomes were not retained by the embryo. A similar observation has been reported in rainbow trout (*Onchrhynchus mykiss*) also [36]. Although it was not possible to unequivocally resolve the mechanism(s) of the observations in our study, it is still clear that at the given microsatellite, there was incomplete retention of maternal or primarily paternal genetic material. Therefore, more than one single mechanism was likely at work.

### Implications for commercial production of triploids

Triploid Atlantic salmon have proven challenging to rear consistently on a commercial scale, but the reasons underpinning their poor performance are not always clear [12, 13, 15]. Consequently, the industry is still almost entirely based on rearing diploid fish. One underlying factor is that the biology and physiology of triploid salmon is different to their diploid siblings [2, 16]. Although some of the production challenges such as cataracts and deformities have been solved with dietary phosphorus and histidine additions [37–40], triploids still occasionally perform poorly in production. These challenges have raised questions of fish welfare in commercial triploid production [13].

Our data demonstrate that pressure-induction of triploidy in Atlantic salmon can result in chromosome aberrations. The frequency of offspring displaying chromosome aberrations was very low in our study, possibly because the triploid induction protocol was diligently adhered to, and consequently, the frequency of triploids was very high. I.e., we did not find evidence of diploids, or what others may have termed as “failed triploids” in our treatment groups. High frequencies of triploids have also been reported in other studies [17, 18]. However, there are also multiple examples directly from the commercial aquaculture industry, including recent examples, whereby flow cytometry has detected batches of fish produced through pressure treatment with triploid frequencies as low as 44% [13]. When this is seen in the light of the results of the present study, and the results of the study in coho salmon that demonstrated that flow cytometry identified diploid coho salmon exposed to pressure treatment are often hyperdiploid aneuploids, i.e., displaying incomplete paternal chromosome retention [17], the possibility opens up that that there are high frequencies of fish displaying diverse chromosome aberrations in batches of triploid Atlantic salmon where triploid induction was accordingly low [13]. In turn, this could contribute to the unexplained poor performance of such groups in the industry. In New Brunswick for example, one batch of triploids had to be terminated as they displayed unexplainably high mortality during start-feeding [15]. Although we did not accurately measure mortality in the present study and can therefore not report it, we detected more extreme inheritance errors in eggs than juveniles sampled at aged 1+, and it is very likely that such extreme cases died between these two sampling points. Therefore, we tentatively suggest that chromosome aberrations may well have been partially responsible for the unexplained high mortality observed during start feeding as reported in New Brunswick [15]. Groups of “triploid” fish have also been terminated in Norwegian aquaculture as a result of unexplained poor performance and in consideration of animal welfare (Lars. H. Stien pers.comm.).

In addition to commercial Atlantic salmon aquaculture, the production and use of triploid fish and shellfish has been trialed over several decades for diverse purposes including reproductive containment of fish released into the wild to control weed growth [41], deliberate augmentation of recreational fisheries without causing genetic changes to the native populations [42, 43], biocontainment of transgenic fish in aquaculture [17] and inhibition of sexual product development to enhance product-value [11]. As within Atlantic salmon aquaculture, other species of triploid fish also display production challenges, and the potential underlying mechanisms reasoned within this study, may also exist for these species. For example, production of triploid Atlantic cod (*Gadus morhua*) has led to poor triploidy induction rates and evidence of increased deformities [44, 45].

### Limitations of the study and future improvements in methodology

This study provides the first genetic examination of inheritance in Atlantic salmon subjected to pressure treatment. Although more labor intensive, genotyping microsatellites provides greater clarity to the challenges associated with triploid induction than the most commonly implemented method of flow cytometry. However, there are still limitations in using microsatellites to detect chromosome aberrations, all of which lead to underreporting of the phenomena [46]. Some of the potential limitations include the following. We only checked genotypes at 16 polymorphic microsatellites, and, have therefore not rigorously examined evidence of inheritance issues on all chromosomes. In some families, parents shared alleles, thus rendering some of the markers “non-informative” with respect to ploidy and inheritance. Also, we were not able to unambiguously distinguish homozygote genotypes as having one, two or three copies.

One of the main limitations in using microsatellites scored by standard (qualitative) analytical procedures is that the alleles are detected, but the numbers of copies of each allele are not. Thus, depending on the mother and/or father’s genotype, even triploid offspring may look like homozygotes or heterozygotes using standard allele calling systems. As stated above, this will lead to an underestimation of inheritance errors [46]. However, where greater precision is required, there are ways to quantify the relative amplification of the alleles to each other, and therefore detect ploidy [47, 48]. Such systems can be challenging to establish for multiple markers, however, future studies of inheritance in Atlantic salmon subjected to hydrostatic pressure treatment could consider using both qualitative as well as quantitative microsatellite genotyping. This would be especially beneficial if a study is performed on a batch of offspring in the industry itself, where the parental genotypes and family backgrounds of the offspring in the tanks or cages are not necessarily available as they were in the present study. Other approaches using amplicon sequencing can also be used to determine ploidy at the genomic level [49, 50], and further developments there may well provide cost-effective approaches in the future.

## Conclusions

### Wider implications and future work

Genetic interactions between domesticated escapees and wild conspecifics has been identified as one of the most significant long-term challenges to an environmentally sustainable Atlantic salmon aquaculture industry [14, 51]. This is also likely to be a situation for other marine species subjected to marine aquaculture [52]. While the proportions of domesticated escapees has declined in Norwegian rivers over the past 2–3 decades [53, 54], suggesting that the situation is getting better, it has been repeatedly suggested that the ultimate solution to stop interbreeding is the production of sterile salmon. Indeed, this has been the primary catalyst for commercial triploid aquaculture [12]. It has also been shown that triploid salmon display reduced migration or return to freshwater following escape from fish farms [55], or release as smolts [56]. Given that domesticated salmon escapees entering rivers are highly frequent carriers of disease-causing agents [57, 58], widespread production of triploid salmon would not only solve direct genetic interactions with wild conspecifics, it would contribute to reduce the possibility of disease interactions also. Therefore, and in the light of the results from the present study, we suggest that greater efforts are needed to investigate the potential link between triploid induction rates, chromosome aberrations and triploid performance in aquaculture. This could be first investigated by looking at microsatellite inheritance patterns and offspring performance in fish that have been subjected to suboptimal pressure treatment, thus deliberately inducting low triploid rates similar to the levels reported on some commercial farms where induction has not been completely successful.

## Methods

### Overall study design

The study is based on three interlinking experiments that involve 32 families, and offspring resulting from the same treatment batches. This includes experiment 1, genotyping 760 eggs from 32 families that had been subjected to hydrostatic pressure treatment and held in separate family units until sampling at the eyed egg stage. Experiment 2, genotyping 1161 communally reared juveniles from 31 of the same 32 families that had been subjected to hydrostatic pressure treatment and were sampled at age 1+. Experiment 3, genotyping 2318 communally reared juveniles from the same 32 families, initially half of which were diploid controls and the other half had been exposed to the same hydrostatic pressure treatment as in experiments 1 and 2.

### Experimental crosses and induction of triploidy

Over a decade, a multiple generation domesticated and wild Atlantic salmon experimental population has been established at the full scale aquaculture facility owned by the Institute of Marine Research in Matre, western Norway [59–64]. These fish have been reared in this facility in a variety of common-garden experiments from start-feeding up to adulthood, and extensive genotyping with microsatellites combined with exclusion based pedigree determination using the program FAP has permitted identification of offspring to their families of origin [65].

In the present study, we generated 32 full and half-sibling families in the following four categories: 8x Domesticated, 8x Domesticated female vs. Wild male, 8x Wild female vs. Domesticated male, 8x Wild. Domesticated broodstock originated from the commercial Mowi strain which is one of the oldest strains used in salmon aquaculture. Broodfish (8 females and 8 males) were stripped for gametes on 22.11.2016 at the Mowi breeding facility on Askøy, western Norway, and transported on ice to IMR's experimental farm in Matre. Simultaneously, wild broodfish (8 females and 8 males) from the river Etne located on the west of Norway [58, 66], were stripped for gametes which were thereafter transported to Matre.

The 32 experimental families were established on the same day as the broodstock were stripped. This followed a standard procedure involving dry fertilisation of eggs with sperm from a single male. Approximately half of these eggs were produced as normal diploids (used only in experiment 3), and the other half were subjected to pressure-induction of triploidy as follows. Precisely 37 min and 30 s after initiation of fertilization at 8 °C, eggs were subjected to hydrostatic pressure of 655 bar for 6 min and 15 s. This procedure has been extensively used in this facility to produce triploid salmonids [40, 67–70]. Eggs were thereafter incubated in single family trays fed by running water at 6 °C. On 09.01.2017 at 289 degree-days, eyed eggs were mechanically shocked. On 11.01.2017, the dead and unfertilized eggs were removed from both the diploid and triploid family groups.

On 02.02.2017 at 436 degree-days, ~ 30 eggs from the pressure-treatment groups for each of the 32 families were sampled into alcohol for genetic analysis. Diploid eggs were unfortunately not sampled at this stage. Only eggs displaying two distinct eyes, indicating normal development up to the stage of sampling, were sampled for genetic analysis. These eggs represent experiment 1. At the same time when experiment 1 was terminated, 40 eggs from 31 of the same 32 families, again only from the pressure treatment, were counted out and mixed into two replicate tanks. These fish represent experiment 2. They were start-fed on 26.04.2017 at a temperature of 13 °C and fed a standard commercial diet in excess. On 15.03.2018, when the fish were approximately 98 g, all were sampled (see results). This including removal of the caudal fin for DNA analysis. Mortality was not recorded at any stage of the study. On the same day that experiment 1 was terminated and experiment 2 was established, 80 eggs from each of the 32 families resulting from both the diploid controls (40 eggs) and pressure treatments (40 eggs) were placed into two mixed ploidy/family replicate tanks. These fish represent experiment 3 and were reared under identical conditions as for the triploid-only fish in experiment 2. They were also terminated and sampled on the same date. Thus, experiment 1 consisted of pressure-treated eggs, experiment 2 consisted of pressure-treated juveniles communally reared, and experiment 3 consisted of both pressure-treated, and untreated diploid controls communally reared.

### Genotyping

In order to assign parents to the juveniles reared in the mixed-family tanks, and to examine inheritance patterns in the eggs and juveniles sampled, all samples were genotyped with a panel of 18 microsatellite markers on an ABI sequencer at the genetics laboratory of the Institute of Marine Research in Bergen, Norway. This panel of microsatellites has been genotyped in this laboratory for more than a decade, involving the analysis of > 50,000 salmon to study population genetics [66, 71, 72], reconstruct pedigree [59, 73, 74], permit identification of conjoined twins [75], conduct forensic investigations to identify the source of escaped salmon back to their farms of origin which also included blind controls and documentation of genotyping error rates [76, 77], and finally, for identification of triploids, trisomic and haploid individuals [5, 7, 55, 69, 78]. Personnel in the laboratory are therefore highly experienced in interpreting genotypes of these markers for a variety of purposes including polyploidy.

DNA was isolated in 96 well format using the DNeasy blood and tissue kit from Qiagen, and each plate contained at least two negative (blank) controls. From eyed eggs, part or whole embryos were dissected out and thereafter used for extraction. For juveniles, caudal fin clips were used. The exact protocols used for PCR amplification of the following 18 microsatellites are available upon request: *SSsp3016* (Genbank no. AY372820), *SSsp2210*, *SSspG7, SSsp2201, SSsp1605, SSsp2216* [79], *Ssa197, Ssa171, Ssa202* [80], *SsaD157, SsaD486, SsaD144* [81], *Ssa289, Ssa14* [82], *SsaF43* [83], *SsaOsl85* [84], *MHC I* [85] *MHC II* [86]. PCR products were amplified on an ABI 3730 Genetic Analyzer and sized by a 500LIZ™ size-standard. Automatically binned alleles in the program Genemapper were independently verified by two researchers prior to exporting data for statistical analyses. Finally, the genotypes of all offspring where inheritance errors were reported (see below) were validated in Genemapper for a third time. Only 16 of the microsatellites were polymorphic and are therefore referred to from this point onwards.

### Determination of ploidy and investigating inheritance patterns

Microsatellites are highly polymorphic genetic markers, often displaying tens of unique alleles within populations. Triploids are thus identifiable by microsatellites as they can display three clearly identifiable alleles per locus [5, 27]. However, not all microsatellite loci will display three distinct alleles in a triploid individual. This will depend on the parental genotypes, as well as the distance of the given microsatellite locus from the centromere and thus the probability of crossing over in the female [87, 88]. A consequence is that even in a true triploid, a limited set of microsatellites may still only reveal up to two alleles per locus, thus making the individual appear as a normal diploid. The use of 16 polymorphic microsatellites as in the present study, however, limits this to a very rare frequency (see Fig. 1 in results). In experiment 3, where diploid controls and pressure-inducted triploids were communally reared from the same families, the above described (but infrequent) phenomena, “failed-triploids” from the pressure-treatment, and/or spontaneous triploids from the diploid controls, have the potential to mix identifications between groups. Despite this theoretical challenge, results coming from experiment 3 were clear and none of these theoretical considerations have influenced data interpretation.

We developed and implemented two overlapping approaches to identify offspring displaying inheritance patterns incompatible with the parental genotypes (see rules below). Offspring displaying errors were first identified in a triploid-inheritance modified version of the exclusion-based family assignment program FAP [65]. This modified version is a fast user-friendly Excel-based program that permits identification of diploid and triploid offspring to their families of origin when the parental genotypes and crosses are known (Supplementary File 3). It reports errors per microsatellite when the offspring’s genotype is in conflict with the parental genotypes according to standard diploid or triploid inheritance and can handle hundreds of families and thousands of offspring simultaneously. It also flags which microsatellite(s) are the cause of the inheritance error, but not the nature of the error. Thereafter, we performed the same analysis in the software language R, using a set of in-house generated scripts (Supplementary File 2). These scripts have the added advantage that they also report the nature of the error (i.e., lacking maternal allele or lacking paternal allele etc.).

On the basis of the results from both approaches described above, offspring genotypes were flagged as having “inheritance errors”, also simply termed “error”, when the offspring displayed one or more of the below conditions for any of the 16 microsatellites analysed:

#### The offspring displayed an allele that was not present in either of the parents

This potentially suggests a mutation in the germline of a parent resulting in a “new” allele in the offspring. Microsatellites display slippage of the repeat motif which is a likely explanation when the new allele is one or two repeats larger or smaller.

#### The offspring lacked an allele(s) from either the mother or the father

This potentially suggests incomplete chromosome retention from either the maternal or paternal line.

#### The offspring displayed more than one allele from the father

This potentially suggests that the offspring inherited a double dose of paternal DNA which is not consistent with the second polar body being retained following pressure treatment.

## Supplementary information

**Additional file 1: Supplementary File 1.** Data.xls, contains all the data associated with this manuscript, including the genotypes (DNA sequences) of all parents and offspring.

**Additional file 2: Supplementary File 2.** R Script.doc, contains the R script used to perform some of the analysis.

**Additional file 3: Supplementary File 3.** FAP_Taggart2007.xlsm, a macro-enabled file containing the family assignment program.

## Data Availability

All data generated or analyzed during this study are included in this published article as supplementary files. The complete data set, including the genotypes (DNA sequences) of all parents and offspring, are attached as an Excel supplementary file 1. The R script used to perform some of the analysis and the family assignment program are attached as a Word supplementary file 2 and an Excel supplementary file 3, respectively.

## Abbreviations

DNA: Deoxyribonucleic acid
PUD: Paternal uniparental diploidy
Bp: Base pair
IMR: Institute of Marine Research
PCR: Polymerase chain reaction
FAP: Family assignment program
