# Supplementary material for: Chromosome aberrations in pressure-induced triploid Atlantic salmon
Source: BMC Genet. 2020 Jun 6;21:59. doi: 10.1186/s12863-020-00864-0 (PMC7276064; doi:10.1186/s12863-020-00864-0)
Supplement: Supplementary file 2 — Additional file 2: Supplementary File 2. R Script.doc, contains the R script used to perform some of the analysis. [file 12863_2020_864_MOESM2_ESM.docx]

##

# checks the pedigree by investigating each possible family against each offspring

source('score_match.R')

l_fam=as.matrix(read.table("families.csv",sep=';',skip=1))

nfam=length(l_fam[,1])

###### import parent data ########

dat_par=as.matrix(read.table("parents_17mark.csv",sep=';',skip=1))

mark_par=as.matrix(read.table("parents_17mark.csv",sep=';',nrows=1))

######## get list of parents markers

mark_par=mark_par[3:length(mark_par)]

temp=NULL

print(mark_par)

for (i in 1:length(mark_par)){

l1=mark_par[i]

l2=unlist(strsplit(l1,'-'))[1]

temp=c(temp,l2)

}

lmark_par=unique(temp)

######################################

### store parent genotypes in list: par_gen

par_gen=list()

l_par=dat_par[,2]

npar=length(dat_par[,1])

nmark_par=length(lmark_par)

for (i in 1:npar){

pari=l_par[i]

par_gen[[pari]]=list()

for (j in 1:nmark_par){

mark=lmark_par[j]

m1=as.integer(dat_par[i,((2*j)+1)])

m2=as.integer(dat_par[i,((2*j)+2)])

par_gen[[pari]][[mark]]=c(m1,m2)

}

}

########

###### import egg data ########

dat_egg=as.matrix(read.table("juvveniles_egg_fam.csv",sep=';',skip=1))

mark_egg=as.matrix(read.table("juvveniles_egg_fam.csv",sep=';',nrows=1))

######## get list of parents markers

mark_egg=mark_egg[4:length(mark_egg)]

temp=NULL

print(mark_egg)

for (i in 1:length(mark_egg)){

l1=mark_egg[i]

l2=unlist(strsplit(l1,'-'))[1]

temp=c(temp,l2)

}

lmark_egg=unique(temp)

######################################

### store egg genotypes in list: egg_gen

egg_gen=list()

l_egg=dat_egg[,3]

negg=length(dat_egg[,1])

nmark_egg=length(lmark_egg)

for (i in 1:negg){

eggi=l_egg[i]

egg_gen[[eggi]]=list()

for (j in 1:nmark_egg){

mark=lmark_egg[j]

m1=as.integer(dat_egg[i,((3*j)+1)])

m2=as.integer(dat_egg[i,((3*j)+2)])

m3=as.integer(dat_egg[i,((3*j)+3)])

egg_gen[[eggi]][[mark]]=c(m1,m2,m3)

}

}

######################################################

#####################################################

#####################################################

#### check all egg against each family

ans=NULL

l_cand=list()

for (i in 1:negg){

res=numeric(nfam)

for (j in 1:nmark_egg){

eggi=l_egg[i]

mark=lmark_egg[j]

G1=egg_gen[[eggi]][[mark]] # get genotype at egg i marker j

for (k in 1:nfam){ # evaluate the nr of error for each possible family

mor=l_fam[k,2]

far=l_fam[k,3]

G2=par_gen[[mor]][[mark]]

G3=par_gen[[far]][[mark]]

temp=score_match(G1,G2,G3)

res[k]=res[k]+as.integer(temp[1]) # need to sum up over all markers !

}

}

cand=NULL

cand=which(res==0) # candidate families are those with zero error

l_cand[[i]]=cand

temp=c(i,res)

ans=rbind(ans,temp) # ans is a matrix (n juveniles X n families) contaning the nr of error for each juvenile / family

}

head=c('*','*','*',mark_egg) # header of output file

egg_fam=as.integer(dat_egg[,2])

outfil='list_juv_inconsist_13122019.csv'

write(head,ncol=length(head),file=outfil,sep=';')

for (i in 1:negg){

eggi=l_egg[i]

if (!(egg_fam[i]%in%l_cand[[i]])){ # if the expected true family is not in the list of candidate families (i.e. at least one error from expected family)

Diag=NULL

write(c(i,egg_fam[i]),file=outfil,append='TRUE',ncol=2)

write(ans[i,],file=outfil,append='TRUE',ncol=length(ans[i,]))

write(' ',file=outfil,append='TRUE')

offg=dat_egg[i,]

mom=l_fam[egg_fam[i],2]

dad=l_fam[egg_fam[i],3]

gmom=par_gen[[mom]]

gdad=par_gen[[dad]]

temp1=mom

temp2=dad

for (mark in lmark_egg){

temp1=c(temp1,gmom[[mark]],'')

temp2=c(temp2,gdad[[mark]],'')

G1=egg_gen[[eggi]][[mark]]

G2=par_gen[[mom]][[mark]]

G3=par_gen[[dad]][[mark]]

temp=score_match(G1,G2,G3)

if (temp[1]==1) Diag=c(Diag,temp[2],'-','-')

if (temp[1]==0) Diag=c(Diag,'-','-','-')

}

Diag=c('-','-','-',Diag)

write(Diag,ncol=length(Diag),file=outfil,append=TRUE,sep=';')

temp1=c('Female','',temp1)

temp2=c('Male','',temp2)

temp_res=rbind(temp1,temp2,offg)

write(t(temp_res),ncol=length(temp1),file=outfil,append=TRUE,sep=';')

write('***',file=outfil,append=TRUE,sep=';')

}

}
